# GTPase domain of porcine Mx1 protein interacts with the N protein of porcine deltacoronavirus to inhibit viral replication

**DOI:** 10.1099/jgv.0.002293

**Published:** 2026-08-05

**Authors:** Haikun Shangguan, Jianxiao Wu, Mingwei Li, Zhaoyang Ji, Yongrui Wang, Chunwei Zhang, Hexin Wang, Dailang Zhong, Zhihong Zhou, Xin Zhang, Da Shi, Hongyan Shi, Longjun Guo, Li Feng, Jianfei Chen

**Affiliations:** 1 Division of Swine Digestive System Infectious Diseases, State Key Laboratory of Animal Disease Control and Prevention, Harbin Veterinary Research Institute, Chinese Academy of Agricultural Sciences, Harbin 150069, PR China

**Keywords:** antiviral activity, interaction, N protein, PDCoV, pMx1, replication

## Abstract

Porcine deltacoronavirus (PDCoV) is an emerging enteric pathogen that poses significant economic threats to the swine industry and carries potential zoonotic risk. However, the interactions between PDCoV and host innate immunity, particularly those involving interferon-stimulated genes (ISGs), remain poorly understood. Porcine myxovirus resistance protein 1 (pMx1), a well-characterized ISG with broad-spectrum antiviral activity, has not been investigated in the context of PDCoV infection. This study demonstrates that PDCoV infection upregulates endogenous pMx1 expression in porcine intestinal epithelial cells (IPEC-J2 and IPI-2I) through a type I interferon alpha-dependent pathway. Functional analyses further revealed that pMx1 exerts potent antiviral activity against PDCoV. Mechanistically, we identified a direct interaction between the pMx1 protein and the PDCoV nucleocapsid (N) protein and found the GTPase domain of pMx1 as the critical binding region. This finding was strongly supported by a predicted interaction complex structural model generated using AlphaFold 3, and the GTPase domain was shown to be indispensable for the antiviral function of pMx1. Furthermore, we identified a critical synergistic interaction between the PDCoV N protein and the viral RNA-dependent RNA polymerase – nonstructural protein 12, a core component of the replication–transcription complex. Co-immunoprecipitation assays demonstrated that pMx1 effectively disrupts this interaction, suggesting that pMx1 may inhibit viral genome replication by impairing the formation or stability of the viral replication complex. Collectively, our study elucidates the mechanism by which the pMx1 protein, as a host antiviral factor, inhibits PDCoV infection. These findings provide novel insights into the innate immune defence against coronaviruses and highlight the pMx1 gene as a promising host-derived antiviral factor with potential applications in antiviral therapeutics and genetic improvement strategies for PDCoV control.

## Data availability

 All data generated or analysed during this study are included in this article.

## Introduction

Since the first coronavirus (CoV), infectious bronchitis virus, was isolated from chicken embryos in 1937 [1], CoVs have remained a major focus of virology research. In recent decades, emerging CoVs, including severe acute respiratory syndrome-associated coronavirus, severe acute respiratory syndrome-associated coronavirus 2 and Middle East respiratory syndrome coronavirus, have posed severe threats to global public health and caused substantial economic losses worldwide [2]. Porcine deltacoronavirus (PDCoV) was first identified in late 2012 in Hong Kong, China [3], and subsequent outbreaks have been reported in multiple countries and regions, including the United States, Canada, South Korea and Thailand [4–7]. PDCoV is a significant swine enteric pathogen capable of infecting and causing disease in pigs of all ages. The virus infects the intestinal epithelial cells of pigs and can lead to acute diarrhoea, vomiting, dehydration and even death in newborn piglets [8]. The clinical manifestations of PDCoV infection are similar to those of other porcine intestinal CoVs, including porcine epidemic diarrhoea virus (PEDV) and transmissible gastroenteritis virus (TGEV) [9]. PDCoV is associated with high morbidity and mortality rates, with fatality rates in neonatal piglets reaching up to 40% [10]. Large-scale outbreaks of PDCoV-induced diarrhoea can, therefore, result in considerable economic losses to the global swine industry. Importantly, accumulating evidence suggests that PDCoV possesses a broader host range than PEDV and TGEV, enabling cross-species infection. Notably, PDCoV RNA has been detected in children with febrile illness in Haiti, highlighting its potential zoonotic risk and raising concerns regarding global public health security [11].

PDCoV is a single-stranded, positive-sense RNA virus. Among all CoVs, deltacoronaviruses possess the smallest genome that encodes four structural proteins – spike (S), nucleocapsid (N), envelope (E) and membrane (M) – as well as a series of nonstructural proteins (Nsps) involved in viral replication and transcription [12]. The N protein is a phosphorylated structural protein that forms the viral nucleocapsid and localizes to both the cytoplasm and nucleolus of infected host cells [13]. As a highly conserved structural protein, the N protein is commonly used as a diagnostic antigen for PDCoV detection. Following viral infection, the N protein is abundantly expressed in host cells and represents one of the earliest viral antigens to induce host antibody responses. Therefore, it serves as an important target for developing therapeutic strategies against PDCoV infection. Similar to other CoVs, PDCoV initiates infection through interaction between its spike (S) protein and host cellular receptors, including aminopeptidase N (APN) [14]. However, recent studies suggest that APN alone is insufficient to explain efficient infection, implying the involvement of additional host factors.

The host’s innate immune response plays a pivotal role in controlling viral infection. IFNs serve as a critical defence mechanism for animals against viruses. Upon viral invasion, IFNs bind to receptors on the surface of neighbouring cells, activating the JAK-STAT signalling pathway [15]. This activation induces the transcription of hundreds of interferon-stimulated genes (ISGs) [16]. The proteins encoded by these genes generally exhibit antiviral activity and play a vital role in the host’s defence against viral infections [17]. Among the numerous antiviral effectors, myxovirus resistance protein 1 (Mx1) has attracted considerable attention due to its potent antiviral activity. Mx1 is an interferon-induced dynamin-like GTPase that is widely distributed among vertebrates, including fish, birds and mammals. The Mx gene was first identified as a dominant antiviral resistance gene in the influenza-resistant inbred mouse strain A2G and was subsequently designated as the murine Mx1 gene [18]. Early studies demonstrated that Mx proteins exhibit strong inhibitory activity against RNA viruses of the *Orthomyxoviridae* family, particularly influenza viruses [19–21]. Subsequent studies further revealed that Mx1 proteins derived from different species display distinct antiviral spectra and that their antiviral specificity is closely associated with their subcellular localization [20, 22].

Significant progress has been made in recent years in elucidating the antiviral mechanisms of the Mx1 proteins. Previous studies have shown that Mx1 inhibits the replication of a broad range of RNA viruses by interfering with viral transcription and nucleocytoplasmic transport. In particular, Mx1 confers resistance to the influenza virus by oligomerizing and trapping viral nucleoproteins [23]. Furthermore, the antiviral activity of Mx1 is closely associated with its GTPase activity and ability to form oligomeric complexes [24]. Although the functions of Mx1 protein have been well characterized in human and murine systems, its role in porcine antiviral defence remains poorly studied. Previous studies have confirmed that porcine myxovirus resistance protein 1 (pMx1) protein exhibits inhibitory effects against several important swine viruses, including classical swine fever virus (CSFV), porcine reproductive and respiratory syndrome virus (PRRSV) and foot-and-mouth disease virus (FMDV) [25–28]. Moreover, our previous work has shown that the interferon-induced transmembrane protein 3 significantly inhibits the attachment phase of viral infection by interacting with the S1 subunit of the PDCoV Spike (S) protein [29]. However, the antiviral role and underlying molecular mechanism of pMx1 against PDCoV infection remain largely unknown.

The interaction between PDCoV and host innate immunity remains incompletely understood. PDCoV employs multiple strategies to evade the host immune response [30], including the modulating IFN signalling pathways [31–33]. However, whether the pMx1 protein acts as a restriction factor against PDCoV and the underlying mechanism involved have yet to be elucidated. Given the structural and functional conservation of pMx1 protein across species, we hypothesized that pMx1 protein may interfere with PDCoV replication through direct viral nucleocapsid binding or by disrupting essential viral replication complexes.

This study aimed to elucidate the antiviral role and underlying mechanism of pMx1 protein in PDCoV infection. Using an *in vitro* PDCoV infection model in porcine intestinal epithelial cell lines (IPEC-J2 and IPI-2I), we hypothesized that pMx1 protein expression is induced during PDCoV infection and inhibits viral replication by targeting key steps. Our results demonstrated that pMx1 protein exhibits significant antiviral activity against PDCoV and the inhibition occurs during the middle to late stages of viral infection. Mechanistically, we identified a direct interaction between pMx1 protein and the PDCoV N protein, with both the antiviral function of pMx1 protein and its interaction with the N protein being dependent on its GTPase domain. More importantly, we discovered that pMx1 protein inhibits the synergistic interaction between the PDCoV N protein and nsp12, the Nsp responsible for viral RNA replication and transcription. Additionally, we employed AlphaFold3 to predict the key interaction interfaces between pMx1 and the PDCoV N protein and experimentally validated the functional importance of these interaction sites in mediating the antiviral activity of pMx1.

## Methods

### Cells and virus

HEK293T cells (human embryonic kidney 293T cells) were purchased from the American Type Culture Collection (CBP60439), IPEC-J2 cells (porcine intestinal columnar epithelial cells) and ST cells (swine testis cells) were maintained in our laboratory and passaged for use by all researchers, IPI-2I cells (porcine ileal epithelial cells) were kindly gifted by Professor Xiao Shaobo of Huazhong Agricultural University [34]. All cell lines were cultured in Dulbecco modified Eagle medium (Gibco, C11995500BT) supplemented with 10% heat-inactivated FBS (Opcell, BS-1101). Cells were grown in a 5% CO_2_ incubator at 37 °C. The PDCoV strain NH (GenBank No.KU981062.1) used in this study was propagated in ST cells. Viral reservoirs were stored at −80 °C, titrated on ST cells and kept in our laboratory.

### Plasmids, antibodies and reagents

The full-length porcine Mx1 gene was amplified from IPEC-J2 cells and cloned into the pCAGGS-FLAG-N vector to obtain a recombinant plasmid (pCAGGS-FLAG-pMx1). Using this plasmid as a template, several pMx1 truncated forms of pMx1 were cloned into the pCAGGS-FLAG-N vector, including pCAGGS-FLAG-GD, pCAGGS-FLAG-GMD and pCAGGS-FLAG-C-pMx1. The codon-optimized S, M, N and nsp12 genes of the PDCoV NH strain (GenBank no. KU981062.1) were cloned into the pcDNA3.1-His (+), pCAGGS-HA and pCMV-Myc vectors to yield the recombinant plasmids pcDNA3.1-S-His, pCAGGS-HA-M, pCAGGS-HA-nsp12 and pCMV-Myc-N. Additionally, we constructed eight mutant pMx1 plasmids using a fast mutagenesis system (TRANSGEN, FM111), including pCAGGS-FLAG-pMx1-A152T, pCAGGS-FLAG-pMx1-V156L, pCAGGS-FLAG-pMx1-G157R, pCAGGS-FLAG-pMx1-R231S, pCAGGS-FLAG-pMx1-E235K, pCAGGS-FLAG-pMx1-D241N, pCAGGS-FLAG-pMx1-D263N and pCAGGS-FLAG-pMx1-R266G. The primers used for cloning and site-directed mutagenesis are listed in Table 1, and all recombinant constructs were verified by sequencing analysis.

**Table 1.** Primers used in the study

Primer	Sequence (5′-3′)
pCAGGS-pMx1-F	CGACGATAAGGAATTCATGGTTTATTCCAGCTGTGAAAGTAAAGAAC
pCAGGS-pMx1-R	GATCTGCTAGCTCGAGTCAGCCTGGGAACTTGGCG
pCAGGS-pMx1-GD-F	CGACGATAAGGAATTCCTGGCCCTGCCCG
pCAGGS-pMx1-GD-R	GATCTGCTAGCTCGAGTCAGGGCAGAGTTTTACAGATG
pCAGGS-pMx1-GMD-F	CGACGATAAGGAATTCGCCCTGCCCGCCAT
pCAGGS-pMx1-GMD-R	GATCTGCTAGCTCGAGTCAGCAGTACACGATCTGCT
pCAGGS-C-pMx1-F	CGACGATAAGGAATTCCTGTTAGAAAACCAAATAAAAGAGAGTCACC
pCAGGS-C-pMx1-R	GATCTGCTAGCTCGAGTCAGCCTGGGAACTTGGCG
pCAGGS-pMx1-A152T-F	GCCCAGATTGCCATCACTGGGGAAGG CGTGGGAATCA
pCAGGS-pMx1-A152T-R	TGATGGCAATCTGGGCTGCGCTGACT TCCTTTTCCA
pCAGGS-pMx1-V156L-F	ATCGCTGGGGAAGGCCTGGGAATCAGTCATGAGCTAATCAGTC
pCAGGS-pMx1-V156L-R	GGCCTTCCCCAGCGATGGCAATCTGGGCTGCGCTGACTTCCTTTTCC
pCAGGS-pMx1-G157R-F	GCTGGGGAAGGCGTGAGAATCAGTCATGAGCTAATC
pCAGGS-pMx1-G157R-R	TCACGCCTTCCCCAGCGATGGCAATCTGGGCTGCGC
pCAGGS-pMx1-R231S-F	ACCACGGAGGCGCTGAGCATGGCCCAGGAGGTGGAC
pCAGGS-pMx1-R231S-R	TCAGCGCCTCCGTGGTGGCAATGTCCACGTTGCAGG
pCAGGS-pMx1-E235K-F	CTGCGCATGGCCCAGAAGGTGGACCCCGAAGGAGACA
pCAGGS-pMx1-E235K-R	TCTGGGCCATGCGCAGCGCCTCCGTGGTGGCAATGT
pCAGGS-pMx1-D241N-F	GTGGACCCCGAAGGAAACAGGACCATCGGGATCTTGAC
pCAGGS-pMx1-D241N-R	TTCCTTCGGGGTCCACCTCCTGGGCCATGCGCAGCG
pCAGGS-pMx1-D263N-F	GAGGACAAGATAGTGAACGTGGCGAGAAACCTGGTCTT
pCAGGS-pMx1-D263N-R	TCACTATCTTGTCCTCAGTGCCTTTGTCCACCAGAT
pCAGGS-pMx1-R266G-F	ATAGTGGACGTGGCGGGAAACCTGGTCTTCCACCTGAA
pCAGGS-pMx1-R266G-R	CCGCCACGTCCACTATCTTGTCCTCAGTGCCTTTGT
q-pMx1-F	CAGAGGCAGCGGAATTGTG
q-pMx1-R	AATCTCGCTGTCCCGGTAAC
q-PDCoV-N-F	AGCAACCACTCGTGTGTTACTTG
q-PDCoV-N-R	CAACTCTGAAACCTTGAGCTG
q-GAPDH-F	ACCTCCACTACATGGTCTACA
q-GAPDH-R	ATGACAAGCTTCCCGTTCTC

The porcine interferon was purchased from PBL Assay Science (17105-1). Mouse monoclonal antibodies against Mx1 were purchased from Bioss (bsm-51528M). Rabbit polyclonal antibodies specific for GAPDH (SAB2108266) were purchased from Thermo. Antibodies against different tags were purchased from Sigma (HA: H6908, Myc: M4439, Flag: F9291). Pierce™ protein A/G magnetic beads were purchased from Thermo Scientific (88803). Lipofectamine RNAiMAX reagent was purchased from Invitrogen (13778150). Alexa Fluor 488 goat anti-rabbit IgG(H+L) (A-11008) and Alexa Fluor 594 goat anti-mouse IgG(H+L) (A-11005) were purchased from Invitrogen. The molecular weight markers (ladder) used in the Western blot experiment were purchased from Thermo Fisher Scientific (26616). Mouse monoclonal antibodies specific for the PDCoV N protein were generated and maintained in our laboratory.

### Western blotting assay

HEK-293T cells, IPEC-J2 cells or IPI-2I cells were lysed in RIPA buffer (Thermo, J63306.AP) supplemented with protease inhibitors (PMSF, 1 : 100). The lysates were incubated on ice for 30 min, then centrifuged at 12,000*g* for 10 min at 4 °C. Cell lysate supernatants were diluted with 5× SDS loading buffer and boiled in boiling water for 10 min. Equal protein samples were separated by SDS-PAGE gel electrophoresis, and gels were transferred to nitrocellulose (NC) membranes (Merck Millipore, HATF85R). The membranes were blocked with 5% skim milk powder, incubated overnight at 4 °C with primary antibodies, washed with PBST buffer and then incubated at 37 °C for 45 min with the corresponding horseradish peroxidase-labelled secondary antibody.

### Real-time fluorescence quantitative PCR

Total RNA was extracted from the collected IPEC-J2 and IPI-2I cells using an RNA extraction kit (BioFlux, BSC52M1). The isolated RNA was then used as a template and reverse transcribed into cDNA using reverse transcriptase (Takara, 6215A). Subsequently, qPCR was performed using SYBR Premix Ex Taq II (Takara, RR820) and the Applied Biosystems Quant Studio 5 Real-Time Fluorescent Quantitative PCR System (Takara) according to the manufacturer’s instructions. Relative gene expression levels were calculated using the 2^(−ΔΔCt) method. The quantitative primers used in this study are listed in Table 1.

### Virus titration assay

Following the overexpression of pMx1 protein in IPEC-J2 and IPI-2I cells, these cells were infected with PDCoV at a m.o.i. of 0.5. Viral supernatants were collected at various time points for TCID_50_ determination using the Reed-Muench method. Specifically, cells were seeded into 96-well plates. Once a confluent monolayer formed, each well was inoculated with 100 µl of a 10-fold serial dilution of the viral suspension. Each dilution was inoculated into eight wells in duplicate. The plates were subsequently incubated for 3–4 days until cytopathic effects (CPEs) were recorded.

### Indirect immunofluorescence assay

Processed IPEC-J2 and IPI-2 I cells were fixed with 4% paraformaldehyde at 4 °C for 30 min, followed by permeabilization with 0.2% Triton X-100 at room temperature for 20 min. Subsequently, cells were blocked with 5% skim milk powder at 4 °C for 1 h, washed three times with PBS and then incubated with mouse monoclonal antibodies against the PDCoV N protein at 37 °C for 1 h. After three PBS washes, cells were incubated with the appropriate FITC-conjugated secondary antibody in the dark at 37 °C for 45 min. After three PBS washes, stain with DAPI at 37 °C in the dark for 15 min. Fluorescence images were subsequently acquired using a laser scanning confocal microscope (Zeiss).

### RNA interference assay

To knock down pMx1 expression in porcine-derived cells, we designed three small interfering RNA (siRNA) duplexes: siRNA-pMx1-1, siRNA-pMx1-2 and siRNA-pMx1-3, which were synthesized by Seven Biotech, and the siRNA duplex target sequences used for pMx1 knockdown are listed in Table 2. According to the manufacturer’s instructions, siRNA was transfected into IPEC-J2 and IPI-2I cells using Lipofectamine RNAiMAX reagent (Invitrogen, 13778150). Cells were harvested 36 h post-infection and analysed by real-time fluorescence quantitative PCR (RT-qPCR).

**Table 2.** The siRNA duplexes used in this study

Name	Sense (5'-3')	Antisense (5'-3')	Length
siRNA-pMx1-1	GGAAGGGCAAAGUCAGUUAdTdT	UAACUGACUUUGCCCUUCCdTdT	21
siRNA-pMx1-2	CAGGGUUUGUGAAUUAUAAdTdT	UUAUAAUUCACAAACCCUGdTdT	21
siRNA-pMx1-3	CGAGAAACCUGGUCUUCCAdTdT	UGGAAGACCAGGUUUCUCGdTdT	21

### Virus adsorption experiment

IPEC-J2 cells, IPI-2I cells and their corresponding pMx1-overexpressing cell lines were seeded into 6-well plates. After 24 h, the cells were pre-chilled at 4 °C, then infected with a high infectious dose of PDCoV (m.o.i.=10) at 4 °C for 1 h. Following infection, the cells were washed three times with pre-chilled PBS to remove unbound virus and then harvested for analysis of viral RNA levels by RT-qPCR.

### Co-immunoprecipitation

Co-transfected HEK293T cells were harvested, washed three times with PBS and lysed with IP lysis buffer (Thermo Scientific, 87787) supplemented with protease inhibitor (PMSF, 1 : 100) at 4 °C for 30 min. The lysate was then centrifuged at 15,000*g* for 10 min at 4 °C. Collect the supernatant, incubate overnight at 4 °C with the indicated antibodies, followed by incubation with an appropriate amount of Protein A/G magnetic beads (Thermo, 88803) according to the manufacturer’s instructions and incubate at 4 °C for 8–10 h. Magnetic beads were then collected using a magnetic stand, washed three times with IP wash buffer and mixed with 5×SDS loading buffer diluted with IP wash buffer. The mixture was heated in boiling water for 10 min before proceeding to Western blot analysis.

### Confocal microscopy

HEK293T cells were seeded in confocal dishes and co-transfected with specific plasmids. After 24 h, cells were fixed with 4% paraformaldehyde at 4 °C for 30 min, permeabilized with 0.2% Triton X-100 at room temperature for 20 min and blocked with PBS containing 5% skim milk for 1 h. The cells were incubated with primary antibody against the FLAG or Myc tag at 37 °C for 1 h, then washed three times with PBST and incubated at 37 °C with Alexa Fluor 488-conjugated goat anti-mouse antibody (Invitrogen, A-11001) or Alexa Fluor 594-conjugated goat anti-rabbit antibody (Invitrogen, A-11012) for 45 min. After washing with PBST, the cells were incubated with DAPI at 37 °C for 15 min. Finally, fluorescence images were acquired using a laser scanning confocal microscope (Zeiss).

### Bioinformatics analysis

The protein sequences of pMx1 (GenBank: NP_999226.2) and PDCoV N protein (GenBank: ALA13749.1) were uploaded to the AlphaFold Server (https://alphafoldserver.com/about). The structure of the complex formed between the pMx1 monomer and the PDCoV N protein was modelled using AlphaFold 3, and the simulation results were visualized with PyMOL software. Low-confidence predicted structures were excluded, resulting in a plausible three-dimensional model of the pMx1-PDCoV N complex.

### Statistical analysis

All experiments in this study were performed in triplicate. Data are expressed as mean±sd. Statistical analysis was performed using GraphPad Prism 8.0 software. A value of *P*<0.05 was considered statistically significant. *P<0.05; **P<0.01; ***P<0.001; ****P<0.0001; ns, not significant.

## Results

### pMx1 expression is induced by type I interferon alpha (IFN-α) and further upregulated in PDCoV-infected IPEC-J2 and IPI-2I cells

**Fig. 1.**
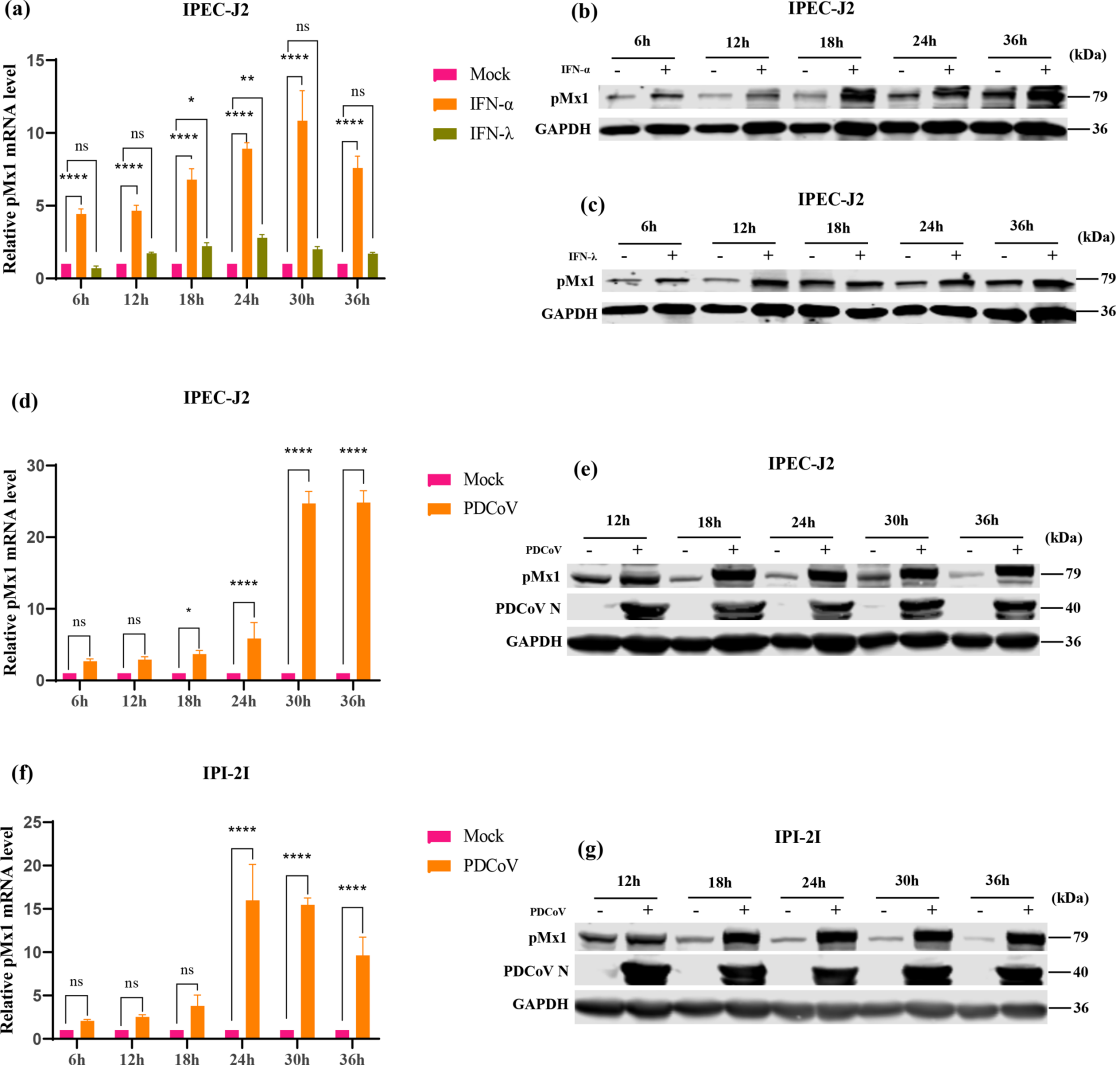
pMx1 is an ISG induced by type I interferon, and PDCoV infection upregulates pMx1 expression in IPEC-J2 cells and IPI-2I cells. IPEC-J2 cells (a–c) were treated with IFN-α or IFN-λ (1.85×10² U), and pMx1 expression levels at different time points, with or without stimulation, were analysed by RT-qPCR and Western blot. IPEC-J2 cells (d, e) and IPI-2I cells (f, g) were infected with PDCoV at an m.o.i. of 0.5, and cell lysates were collected at the indicated time points post-infection. Changes in pMx1 mRNA and protein levels were determined by RT-qPCR and Western blot. Data are presented as the mean±sd of three independent experiments. Statistical significance was analysed using two-way ANOVA followed by Bonferroni’s post-hoc test between IFN-treated groups and the mock control. *P<0.05; **P<0.01; ****P<0.0001; ns, not significant.

We confirmed that the porcine Mx1 gene is an ISG induced by type I interferon alpha (IFN-α), and that PDCoV infection upregulates the endogenous expression of pMx1 in IPEC-J2 and IPI-2I cells, respectively. In addition, we experimentally confirmed that PDCoV infection can induce the production of IFN-α, which occurs at the late stage of infection, whereas IFN-α levels are suppressed during the early stage of infection (Fig. S1, available in the online Supplementary Material). Specifically, IPEC-J2 cells were treated with porcine interferon IFN-α and IFN-λ (activity: 1.85×10^2^ U), with a blank control group established. Cells from both experimental and control groups were collected every 6 h, and endogenous pMx1 mRNA levels (Fig. 1a) and protein levels (Fig. 1b, c) were detected by RT-qPCR and Western blot. We found that after IFN-α stimulation, the mRNA and protein levels of pMx1 in the experimental group were significantly upregulated compared to the control group, while the effect of IFN-λ was less pronounced than that of IFN-α. Subsequently, IPEC-J2 and IPI-2I cells were infected with PDCoV (m.o.i.=0.5), and cell lysates were collected at specific time points. Changes in pMx1 mRNA and protein levels were detected by RT-qPCR and Western blot. The results showed that pMx1 mRNA levels began to increase significantly at 24 h post-infection (Fig. 1d, f), while pMx1 protein levels in the experimental group remained higher than those in the control group (Fig. 1e, g).

### Overexpression of pMx1 inhibits replication of PDCoV

**Fig. 2.**
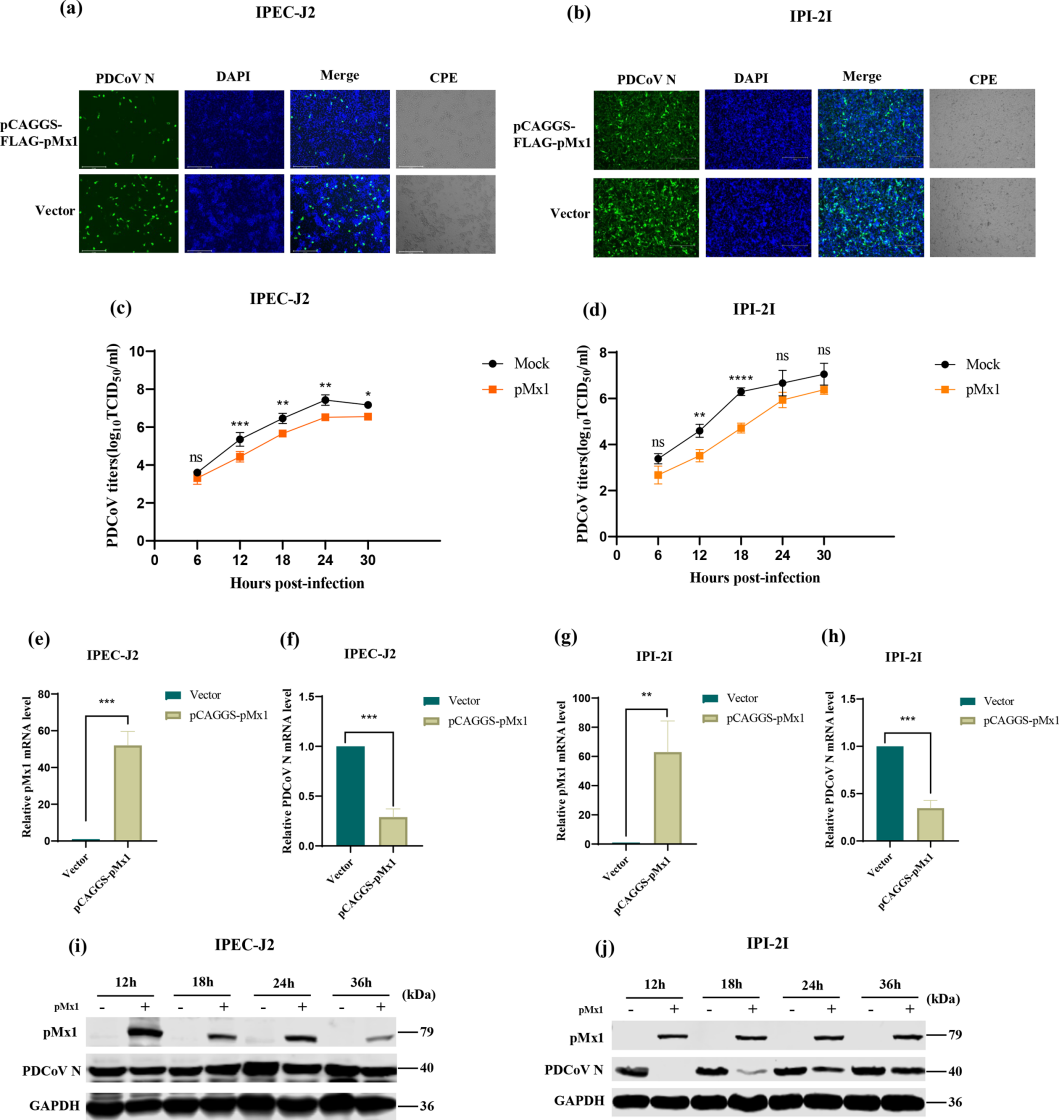
(a, b) IPEC-J2 and IPI-2I cells were seeded in 12-well plates and transfected with equal amounts of the pMx1 expression plasmid (pCAGGS-N-Flag-pMx1). At 24 h post-transfection, cells were infected with PDCoV. After 24 h of infection, cells were fixed with 4% paraformaldehyde, and PDCoV infection levels were evaluated by IFA and CPE observation. (c, d) Following pMx1 overexpression in IPEC-J2 and IPI-2I cells, cells were infected with PDCoV, and supernatants were collected at the indicated time points to determine viral titres (log-transformed values; mean±sd; *n*=3). Statistical significance was analysed using two-way ANOVA followed by Bonferroni’s post-hoc test between pMx1-overexpressing groups and the mock control. *P<0.05; **P<0.01; ***P<0.001; ****P<0.0001; ns, not significant. (e–j) IPEC-J2 and IPI-2I cells were transfected with the pMx1 expression plasmid and subsequently infected with PDCoV. Cell lysates were collected at the indicated time points post-infection, and changes in PDCoV N mRNA and protein levels were analysed by RT-qPCR and Western blot. Data are presented as the mean±sd of three independent experiments. Statistical analysis was performed using a two-tailed unpaired Student’s t-test. **P<0.01; ***P<0.001.

To determine whether pMx1 exhibits antiviral activity against PDCoV, a series of validation experiments were performed. Briefly, IPEC-J2 and IPI-2I cells were transiently transfected with the pCAGGS-FLAG-pMx1 plasmid to overexpress pMx1, while empty vector (Vector) transfection served as the control. Twenty-four hours post-transfection, cells were infected with PDCoV at an m.o.i. of 1. After 24 h of infection, indirect immunofluorescence assays (IFAs) were performed to assess PDCoV infection levels based on fluorescence intensity and CPEs. The results showed that in IPEC-J2 cells and IPI-2I cells (Fig. 2a, b), the fluorescence intensity in the transfection group was significantly lower than that in the control group. Correspondingly, the CPE in the control group was more severe than that in the transfection group.

Subsequent experiments involved overexpressing pMx1 in IPEC-J2 and IPI-2I cells, followed by PDCoV infection. Briefly, pMx1 was overexpressed in IPEC-J2 and IPI-2I cells prior to PDCoV infection, and viral supernatants were collected at the indicated time points to determine viral titres. The results demonstrated that viral titres peaked at ∼24 h post-infection. Notably, in both IPEC-J2 and IPI-2I cells, the viral titres in the transfection group were consistently lower than those in the control group (Fig. 2c, d), indicating that pMx1 overexpression suppresses PDCoV infection to some extent. We further conducted pMx1 overexpression in both cell lines prior to PDCoV inoculation. Post-infection cell samples were collected, and changes in PDCoV N mRNA and protein levels were detected by RT-qPCR and Western blot, respectively. The results revealed significantly lower levels of both PDCoV N mRNA and protein in the transfection group compared to the control group (Fig. 2e–j), further confirming that pMx1 overexpression exerts inhibitory effects on PDCoV infection. Notably, the inhibitory effect of pMx1 on PDCoV was more pronounced in IPI-2I cells than in IPEC-J2 cells.

### Knocking down the expression of pMx1 promotes the replication of PDCoV

**Fig. 3.**
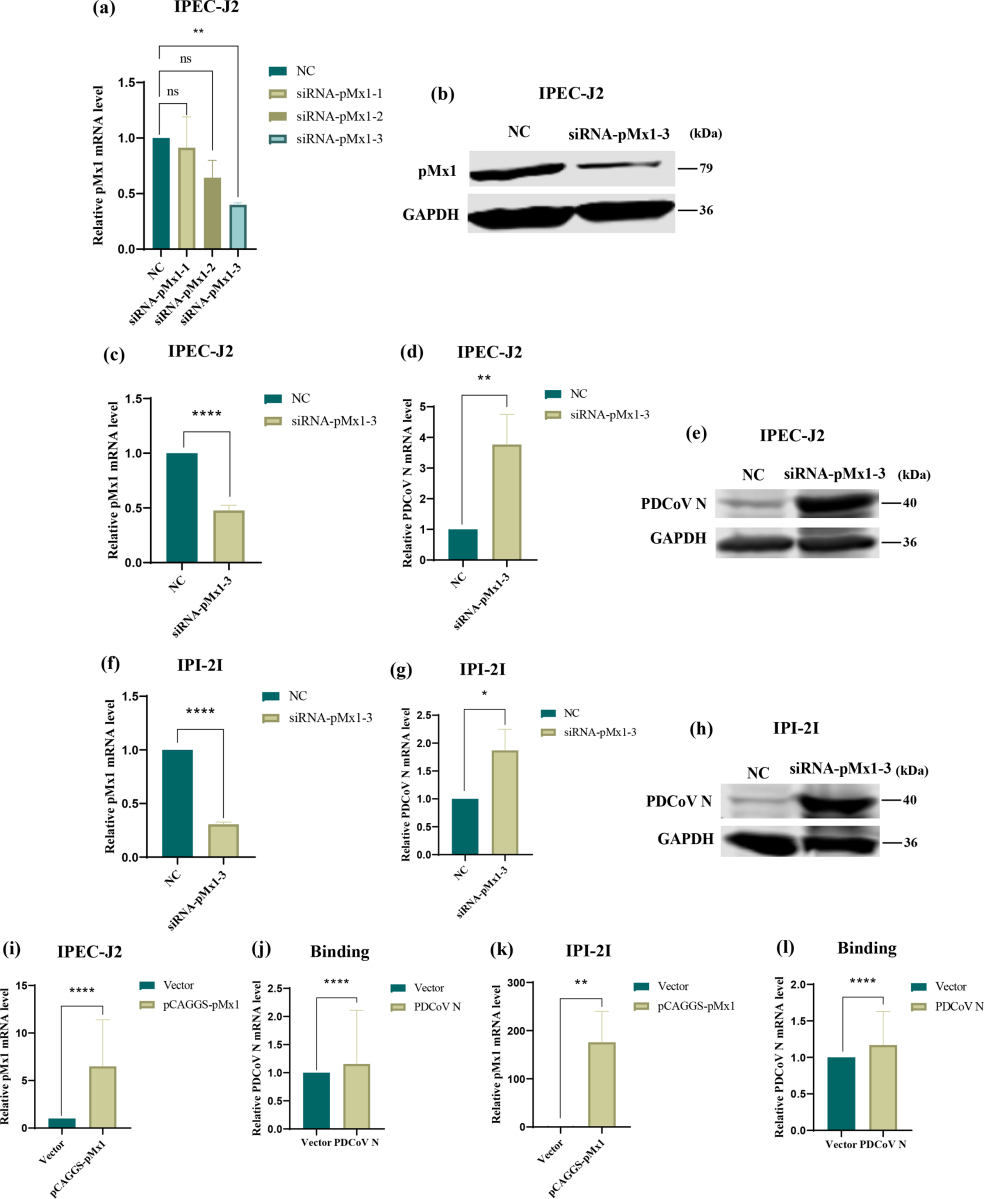
(a, b) Three siRNAs targeting pMx1 were designed. The siRNA showing the highest knockdown efficiency was selected based on RT-qPCR analysis and further confirmed by Western blot. Data are presented as the mean±sd of three independent experiments. Statistical significance was analysed using one-way ANOVA with Dunnett’s post-hoc test. **P<0.01; ns, not significant. (c–h) IPI-2I and IPEC-J2 cells were transfected with siRNA-pMx1-3 or negative control (NC), followed by infection with PDCoV (m.o.i.=0.5). At 24 h post-infection, the mRNA and protein levels of PDCoV N were analysed by RT-qPCR and Western blot. (i, l) IPI-2I and IPEC-J2 cells were transfected with the pMx1 expression plasmid in 12-well plates. At 24 h post-transfection, cells were pre-chilled for 30 min and then incubated with PDCoV (m.o.i.=10) at 4 °C for 1 h to allow viral attachment. Cells were washed three times with pre-cooled PBS to remove unbound virus, and intracellular viral RNA levels during the attachment stage were determined by RT-qPCR. Data are presented as the mean±sd of three independent experiments. Statistical analysis was performed using a two-tailed unpaired Student’s t-test. *P<0.05; **P<0.01; ****P<0.0001.

To further investigate the impact of endogenous pMx1 protein on PDCoV infection, we performed a loss-of-function experiment using RNA interference. Three siRNAs targeting pMx1 were designed and transfected into IPEC-J2 and IPI-2I cells to suppress endogenous pMx1 expression. Among them, siRNA-pMx1-3 was selected as the most effective construct based on RT-qPCR screening (Fig. 3a), achieving more than 50% knockdown efficiency, which was further confirmed by Western blot analysis (Fig. 3b). Subsequently, IPI-2I and IPEC-J2 cells were transfected with siRNA-pMx1-3 or control siRNA (NC) followed by PDCoV infection, respectively. The expression levels of PDCoV N mRNA and protein were detected by RT-qPCR and Western blot, respectively. The results demonstrated that the viral RNA load in the siRNA-pMx1-3 group was approximately two to fourfold higher than in the NC group (Fig. 3c–h), indicating that knockdown of pMx1 expression significantly promoted PDCoV infection.

### The pMx1 protein does not affect the attachment phase of PDCoV infection

To investigate whether the pMx1 protein functions during the attachment phase of PDCoV infection, IPEC-J2 and IPI-2I cells were seeded in 12-well plates and transfected to overexpress pMx1, respectively. After 24 h post-transfection, the cells were pre-cooled for 30 min and then infected with a high infectious dose of PDCoV (m.o.i.=10) at 4 °C for 1 h. Subsequently, the cells were washed three times with pre-chilled PBS and collected for viral mRNA detection by RT-qPCR. RT-qPCR analysis revealed no significant difference in viral RNA levels between pMx1-overexpressing cells and control cells (Fig. 3i–l), suggesting that pMx1 protein does not influence the attachment phase of PDCoV infection.

### The PDCoV N protein interacts with pMx1 and co-localizes in the perinuclear cytoplasm

**Fig. 4.**
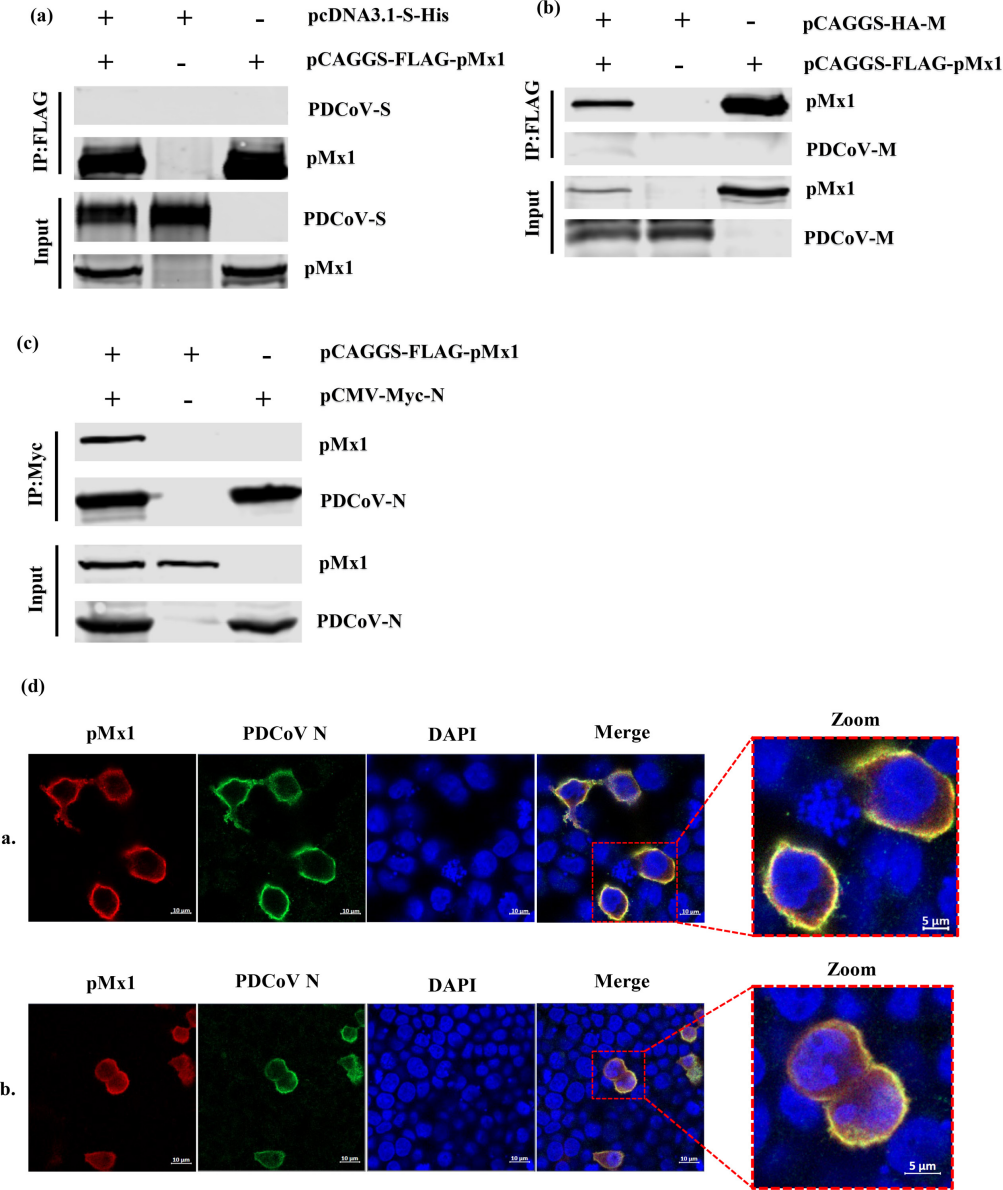
The PDCoV N protein interacts with pMx1 and co-localizes in the perinuclear cytoplasm. (**a–c**) In HEK293T cells, His-PDCoV S was co-transfected with FLAG-pMx1, Myc-PDCoV N was co-transfected with FLAG-pMx1, and HA-PDCoV M was co-transfected with FLAG-pMx1; transfection of His-PDCoV S, HA-PDCoV M, Myc-PDCoV N, or FLAG-pMx1 alone was left as a control. At 36 h post-transfection, the cells were collected and lysed for co-immunoprecipitation (Co-IP) analysis with anti-FLAG (**a, b**) or anti-Myc (**c**) antibodies, respectively. (**d**) In HEK 293 T cells, FLAG-pMx1 and Myc-PDCoV N were co-transfected, while FLAG-pMx1 or Myc-PDCoV N alone were transfected as controls. Immunofluorescence staining was then performed, and cells were examined by confocal microscopy to determine the localization of PDCoV N protein (green), pMx1 (red), and the nucleus (blue).

This study has confirmed that pMx1 exerts an inhibitory effect on PDCoV infection, yet the underlying mechanisms and its direct or indirect interactions with PDCoV structural proteins remain unclear. To address this, we screened PDCoV structural proteins – specifically the S, M and N proteins – for potential interactions with pMx1. We co-transfected HEK293T cells with pairwise combinations: PDCoV S and pMx1, PDCoV N and pMx1 and PDCoV M and pMx1, respectively. Co-IP assays demonstrated a specific interaction between pMx1 and the PDCoV N protein, whereas no detectable interaction was detected with either the S or M proteins (Fig. 4a–c). To further validate this finding, we co-transfected HEK293T cells with pMx1 and the PDCoV N protein and performed confocal microscopy to examine their subcellular colocalization. The results revealed that pMx1 and the PDCoV N protein colocalized primarily in the perinuclear region of the cytoplasm (Fig. 4d).

### GTPase domain of pMx1 interacts with the PDCoV N protein

**Fig. 5.**
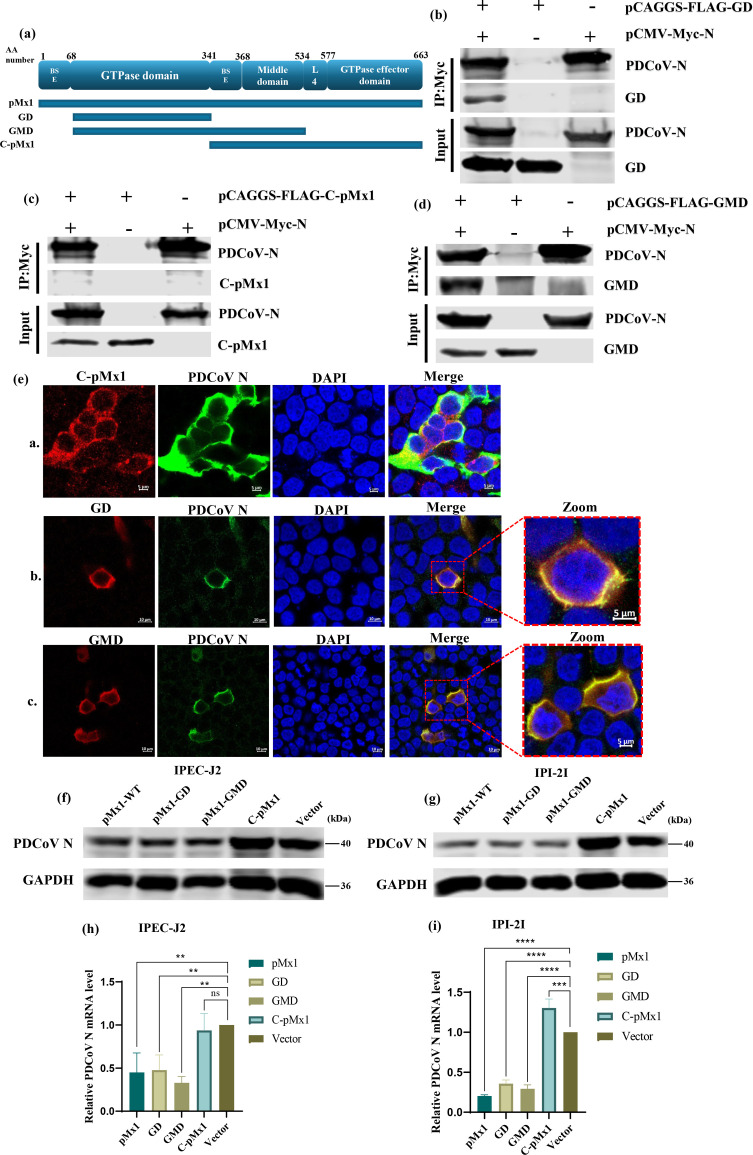
The GTPase domain of pMx1 is required for its interaction with PDCoV N. (**a**) Schematic diagram of pMx1 protein domains and the positions of the truncation mutants used in this study. (b–d) HEK293T cells were co-transfected with Myc-PDCoV N and FLAG-tagged pMx1 truncation constructs (FLAG-GD, FLAG-C-pMx1, or FLAG-GMD). Cells transfected with FLAG-GD, FLAG-C-pMx1, FLAG-GMD or Myc-PDCoV N alone served as controls. At 36 h post-transfection, cells were harvested and lysed for Co-IP assays using anti-Myc antibodies, as indicated. (**e**) Subcellular localization of PDCoV N and the pMx1 truncation mutants in HEK293T cells was examined by confocal microscopy following co-transfection. (F–I) Equal amounts of full-length pMx1, its truncation mutants, or empty vector were transfected into IPEC-J2 and IPI-2I cells. At 24 h post-transfection, cells were infected with PDCoV (m.o.i.=0.5). At 24 h post-infection, the mRNA and protein levels of PDCoV N in cell lysates were analysed by RT-qPCR and Western blot. Data are presented as the mean±sd of three independent experiments. Statistical significance was analysed using one-way ANOVA with Dunnett’s post-hoc test. **P<0.01; ***P<0.001; ****P<0.0001; ns, not significant.

To define the domain of pMx1 responsible for its interaction with the PDCoV N protein, a series of truncation mutants were constructed, including pCAGGS-FLAG-GD, pCAGGS-FLAG-GMD and pCAGGS-FLAG-C-pMx1 (Fig. 5a). Following co-transfection with the PDCoV N expression plasmid in HEK293T cells, Co-IP assays demonstrated that only pMx1 variants containing an intact GTPase domain were capable of interacting with the PDCoV N protein (Fig. 5b–d). Consistently, confocal microscopy demonstrated robust colocalization of PDCoV N with GTPase domain – containing truncations, whereas mutants lacking this domain showed no appreciable colocalization (Fig. 5e).

To determine whether the GTPase domain is required for the antiviral function of pMx1, full-length pMx1 and its truncation mutants were overexpressed in IPEC-J2 and IPI-2I cells prior to PDCoV infection. RT-qPCR and Western blot analyses at 24 h post-infection showed that only cells expressing full-length pMx1 or GTPase domain – containing truncations exhibited a significant reduction in PDCoV N mRNA and protein levels compared with the empty vector control (Fig. 5f–i). Notably, pMx1-GD and pMx1-GMD retained antiviral activity comparable to that of WT pMx1, whereas the C-terminal fragment (C-pMx1), which lacks the GTPase domain, largely lost its inhibitory capacity. Intriguingly, in IPI-2I cells, expression of C-pMx1 appeared to increase PDCoV N protein levels relative to the control, suggesting a potential dominant-negative or modulatory effect.

### pMx1 weakens the synergistic interaction between PDCoV N protein and PDCoV nsp12

**Fig. 6.**
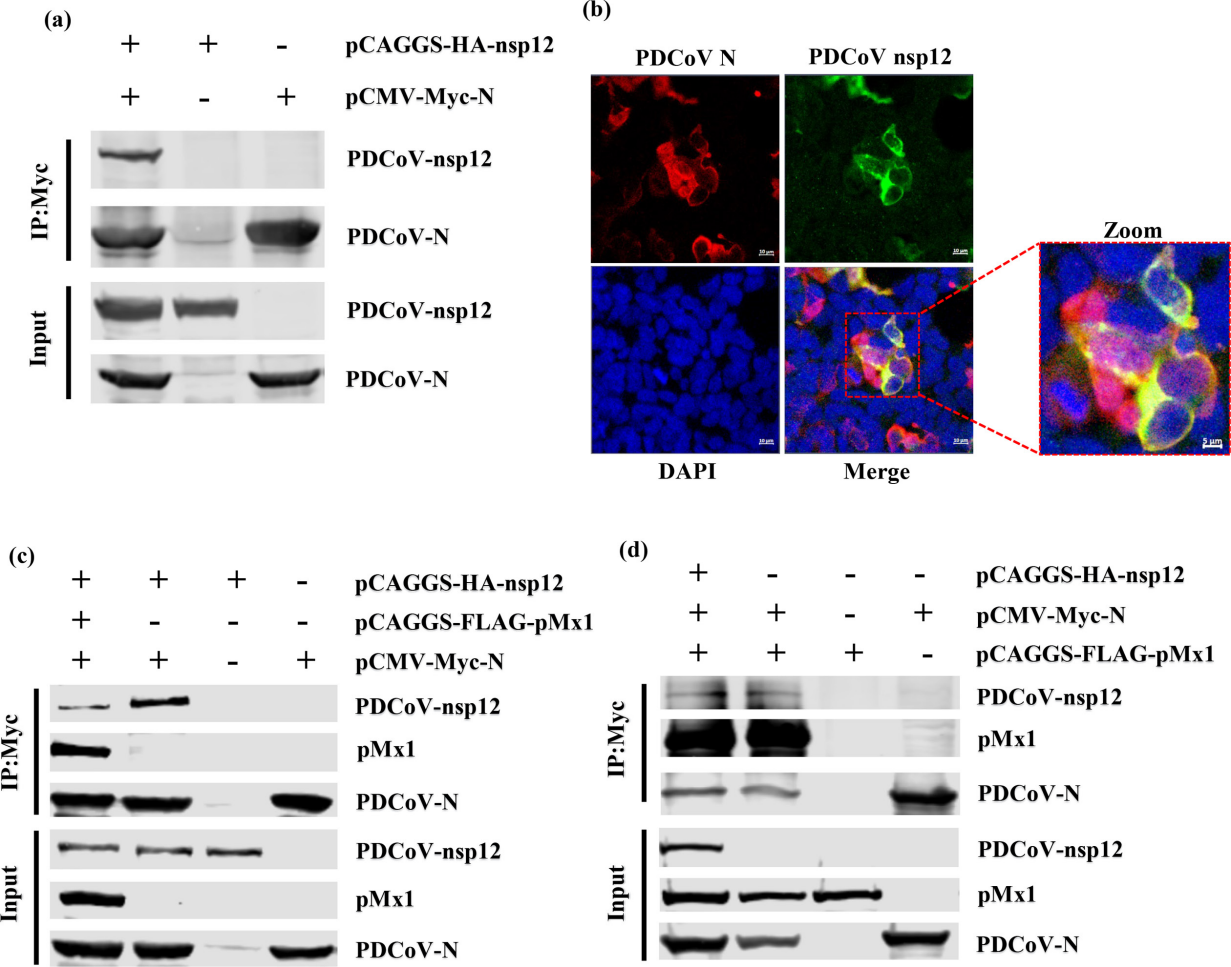
PDCoV N interacts with nsp12 and co-localizes with it in the perinuclear region, and pMx1 attenuates their interaction. (**a**) HEK293T cells were co-transfected with HA-nsp12 and Myc-PDCoV N expression plasmids. Cells transfected with HA-nsp12 or Myc-PDCoV N alone served as controls. At 36 h post-transfection, cells were harvested and lysed for Co-IP assays using anti-Myc antibodies, as indicated. (**b**) HEK293T cells were co-transfected with PDCoV N and nsp12 expression plasmids, and their subcellular localization was analysed by confocal microscopy. (c, d) HEK293T cells were co-transfected with FLAG-pMx1, HA-nsp12 and Myc-PDCoV N expression plasmids. Co-IP assays were performed to evaluate (**c**) the effect of pMx1 on the interaction between PDCoV N and nsp12, and (**d**) the influence of nsp12 on the interaction between pMx1 and PDCoV N.

A complex interaction network between viral structural and Nsps plays a critical role in regulating viral replication, immune evasion and pathogenicity [35]. To determine how pMx1 affects post-entry PDCoV infection and to identify viral Nsps that functionally associate with the PDCoV N protein, we screened candidate PDCoV Nsps for potential interaction with N using Co-IP assays. Among the proteins tested, PDCoV nsp12 exhibited a robust interaction with the N protein (Fig. 6a). Confocal microscopy further demonstrated that PDCoV N and nsp12 predominantly colocalized in the perinuclear region of HEK293T cells (Fig. 6b), consistent with their potential cooperation within the replication–transcription complex (RTC).

To investigate whether the antiviral activity of pMx1 is linked to modulation of the N–nsp12 interaction, we assessed both the effect of pMx1 on the association between PDCoV N and nsp12, and the influence of nsp12 on the pMx1–N interaction. HEK293T cells were co-transfected with expression plasmids encoding pMx1, PDCoV N and PDCoV nsp12, followed by Co-IP analysis. The results revealed that the presence of pMx1 markedly reduced the interaction between PDCoV N and nsp12 (Fig. 6c). In contrast, nsp12 did not affect the interaction between pMx1 and PDCoV N (Fig. 6d), indicating that pMx1 selectively disrupts the N–nsp12 complex while maintaining its binding to the N protein.

### The predicted key interaction sites between pMx1 and PDCoV N protein are located within the GTPase domain of pMx1

**Fig. 7.**
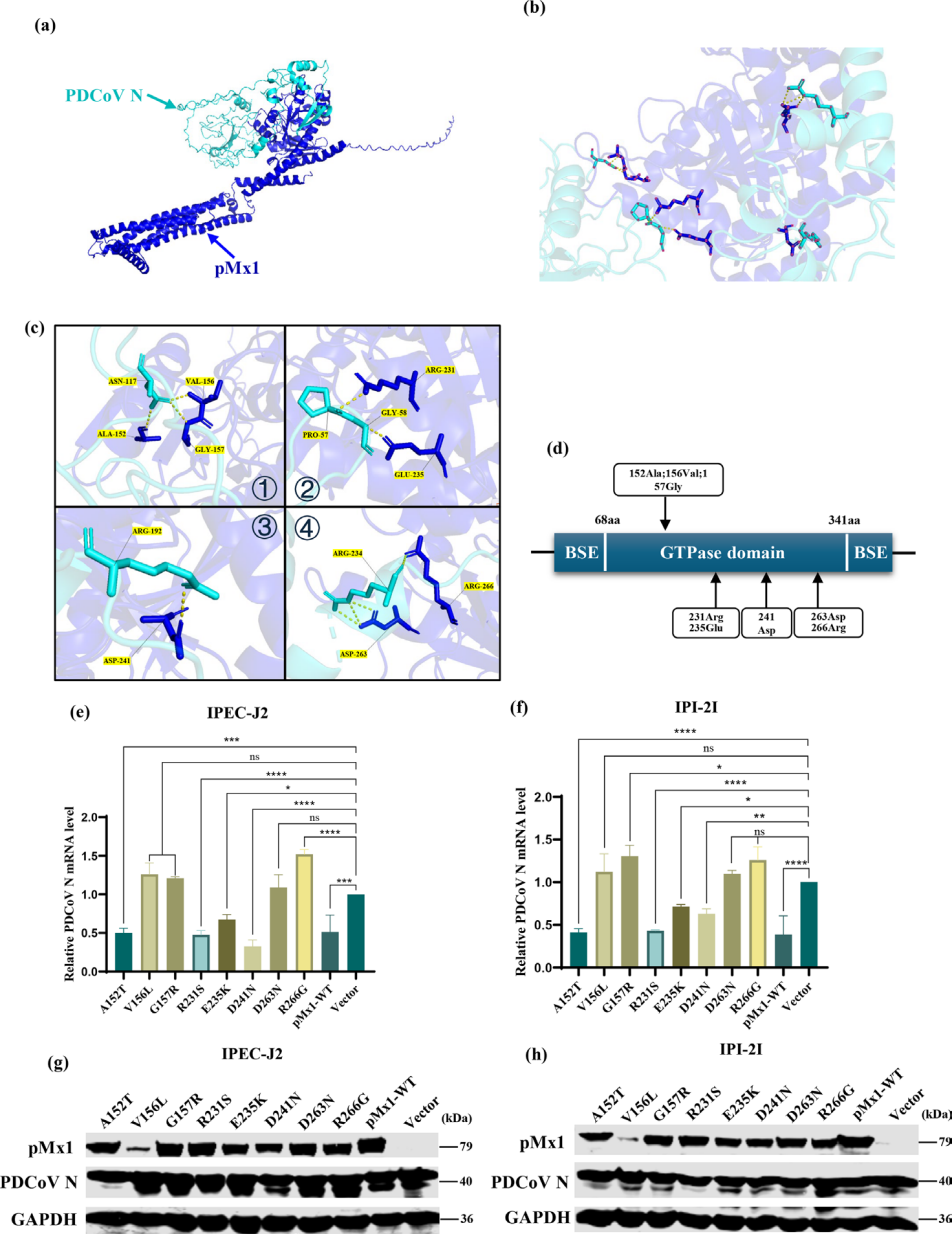
The predicted interface between pMx1 and PDCoV N is located within the GTPase domain of pMx1. (**a**) Three-dimensional structural model of the complex formed by the pMx1 monomer and PDCoV N protein, predicted using AlphaFold three and visualized with PyMOL. The pMx1 monomer is shown in dark blue, and PDCoV N is shown in light blue. (**b**) Enlarged view highlighting the predicted interaction interface between pMx1 and PDCoV N. (**c**) Structural domain annotations and specific amino acid residues involved in the predicted interaction between the pMx1 monomer and PDCoV N. (**d**) Schematic representation of the relative positions of the predicted interaction residues within the pMx1 protein. (e–h) WT pMx1 and eight pMx1 point mutants were transfected into IPEC-J2 and IPI-2I cells. At 24 h post-transfection, cells were infected with PDCoV (m.o.i.=0.5). PDCoV N mRNA and protein levels in cell lysates were analysed by RT-qPCR and Western blot. Data are presented as the mean±sd of three independent experiments. Statistical significance was analysed using one-way ANOVA with Dunnett’s post-hoc test. *P<0.05; **P<0.01; ***P<0.001; ****P<0.0001; ns, not significant.

To gain structural insight into the interaction between pMx1 and the PDCoV N protein, we employed AlphaFold 3 to predict the structure of the pMx1–PDCoV N complex. The predicted model exhibited high confidence and revealed a well-defined interaction interface (Fig. 7a). Structural analysis identified four distinct interaction regions between pMx1 and PDCoV N (Fig. 7b). Eight key amino acid residues in pMx1 were predicted to participate in intermolecular interactions (Fig. 7c), and these residues are summarized in Table 3. Notably, all identified residues are located within the GTPase domain of pMx1 (Fig. 7d).

**Table 3.** Predicted interaction sites between pMx1 and PDCoV N protein

Interaction domain	Residue no. of pMx1	Residue name	Residue no. of PDCoV N	Residue name
**①**	152,156,157	Ala, Val, Gly	117	Asn
**②**	231,235	Arg, Glu	57,58	Pro, Gly
**③**	241	Asp	192	Arg
**④**	263,266	Asp, Arg	234	Arg

To functionally validate the predicted interaction residues, we generated eight pMx1 point mutants targeting the corresponding amino acid positions. These mutants, along with WT pMx1 (pMx1-WT) and the empty vector control, were transfected into IPEC-J2 and IPI-2I cells, followed by PDCoV infection. At 24 h post-infection, PDCoV N mRNA and protein levels in cell lysates were analysed by RT-qPCR and Western blot, respectively. RT-qPCR results showed that the V156L, G157R, D263W and R266G mutants largely lost their ability to suppress PDCoV replication. Western blot analysis further revealed that the antiviral activity of mutants V156L, G157R, E235K, D263W and R266G was impaired in IPEC-J2 cells, whereas the reduction in antiviral activity was less pronounced in IPI-2I cells (Fig. 7e–h).

## Discussion

The host innate immune system, particularly the interferon-induced response, serves as the first line of defence against viral infections [15]. Previous studies have revealed that the host factor pMx1 protein exerts antiviral effects against several viruses, including PRRSV, CSFV and FMDV, suggesting its broad-spectrum antiviral potential. However, the mechanism of pMx1 against PDCoV has not been clearly reported. In this study, we demonstrate that pMx1 significantly inhibits PDCoV replication in IPEC-J2 and IPI-2I cells and provide preliminary insights into the underlying mechanism of action.

Our findings confirm that PDCoV infection can induce IFN-α expression, predominantly during the later stages of infection, while its expression is partially suppressed in the early phase. Previous related studies have reported similar conclusions [16]. We also found that IFN-α is a key factor in inducing the elevation of endogenous pMx1 levels in IPEC-J2 and IPI-2I cells. Previous studies have also demonstrated that IFN-α levels are positively correlated with Mx1 induction [36], which aligns perfectly with the conclusions drawn in our research. The observed function of pMx1 is consistent with the established paradigm of Mx1 as a classic ISG, indicating that cells deploy this antiviral effector to combat PDCoV infection. Through both pMx1 overexpression and knockdown experiments, its inhibitory effect on PDCoV infection was unequivocally demonstrated. pMx1 overexpression significantly suppressed PDCoV replication, whereas knockdown of endogenous pMx1 enhanced viral proliferation. These mutually supporting experimental results provide compelling evidence that pMx1 serves not merely as a biomarker of IFN-α activation, but as a key executor of the antiviral state against PDCoV, effectively limiting viral infection in IPEC-J2 and IPI-2I cells, respectively.

The PDCoV N protein protects the viral genome by encapsulating viral nucleic acids to form the viral nucleocapsid, playing critical roles in viral RNA synthesis and virion assembly [37]. Accordingly, the N protein predominantly functions during the middle and late stages of viral infection. In our study, we demonstrated that pMx1 interacts with the PDCoV N protein to inhibit viral replication. Through viral attachment assays, we confirmed that pMx1 expression levels do not affect the early stage of PDCoV infection, further confirming that pMx1-mediated inhibition of PDCoV replication through targeting the N protein occurs during the middle and late phases of the viral life cycle.

The pMx1 protein belongs to the dynamin superfamily and is composed of 663 amino acids, mainly including an N-terminal GTPase domain, a middle domain and a C-terminal effector domain [24]. The GTPase domain is responsible for GTP binding and hydrolysis, thereby conferring GTPase activity to the pMx1 molecule [38]. As a consensus motif of the Mx protein family, the GTPase domain is crucial for pMx1 function. Studies on the antiviral effects of pMx1 against pseudorabies virus and PRRSV have demonstrated that its GTPase activity plays a key role in inhibiting viral infection [26, 39]. In addition, several antiviral mechanisms of pMx1 have been reported. For example, pMx1 can interfere with the early endocytic pathway of influenza A virus, thereby impeding viral entry into the nucleus and thereby suppressing replication [40]. In another study, co-expression of pMx1 with the internal ribosome entry site of FMDV significantly enhanced its antiviral activity against FMDV in PK-15 cells [27]. Additionally, a fusion protein created by combining pMx1 with the Human immunodeficiency virus transduction peptide domain was shown to markedly inhibit infections by CSFV and vesicular stomatitis virus [41, 42]. Through Co-IP assays and site-directed mutagenesis of key residues, our study established that the GTPase domain of pMx1 serves not only as the critical structural domain for its interaction with the PDCoV N protein but also as the fundamental basis for its antiviral function. These findings suggest that the anti-PDCoV activity of pMx1 is likely dependent on its GTP-binding or GTP-hydrolyzing activity.

In virus-infected cells, the N protein may localize exclusively in the cytoplasm or simultaneously in both the cytoplasm and nucleus [43]. Previous studies indicate that PDCoV RNA synthesis and ribosome assembly are facilitated through interactions between the N protein and ribosomal subunits or nucleolar proteins [13]. All positive-strand RNA viruses encode an RNA-dependent RNA polymerase (RdRp), which cooperates with both host and viral factors to mediate viral genome replication [44]. In PDCoV, nsp12 functions as the RdRp responsible for viral RNA replication and transcription. As a core component of viral replication, nsp12 plays a critical role in viral nucleic acid synthesis. Based on these findings, we investigated whether the PDCoV N protein and nsp12 exhibit functional cooperation during viral genome replication and packaging. Using Co-IP assays, we identified an interaction between the N protein and nsp12, suggesting that the N protein may engage with the viral RTC, which is primarily organized by nsp12 and other viral proteins and is essential for efficient viral replication. Furthermore, we demonstrated that pMx1 attenuates this critical synergistic interaction between the PDCoV N protein and nsp12. This disruption likely compromises the integrity and functionality of the RTC, consequently leading to significant inhibition of viral RNA synthesis. This finding highlights a sophisticated antiviral mechanism of pMx1 that targets a key step in the CoV life cycle.

The predicted structure of the pMx1-PDCoV N protein interaction complex revealed that several key amino acid residues within the GTPase domain form hydrogen bonds and hydrophobic interactions with specific residues on the surface of the PDCoV N protein. These structural predictions are consistent with our experimental findings and strongly support the conclusion that the GTPase domain functions as the effector domain responsible for direct binding to the viral N protein. It also offers a plausible structural explanation for the ability of pMx1 to disrupt the N-nsp12 interaction. Specifically, binding of the pMx1 GTPase domain to the PDCoV N protein may create steric hindrance or induce conformational changes in the N protein, thereby preventing its proper association with nsp12. Disruption of this interaction is likely to compromise the integrity and functionality of the RTC, ultimately interfering with viral nucleic acid replication.

In summary, our study confirms that pMx1 effectively inhibits PDCoV infection and elucidates a multi-layered antiviral mechanism against this pathogen. Specifically, pMx1 directly interacts with the PDCoV N protein through its GTPase domain. This interaction disrupts the functional association between the N protein and the viral RNA polymerase nsp12, thereby suppressing viral genomic RNA synthesis and ultimately suppressing viral replication. Collectively, our work identifies pMx1 as a significant restriction factor for PDCoV, advances our understanding of the interactions between PDCoV and host factors and provides novel insights into the innate immune defence mechanisms against PDCoV infection.

## Supplementary material

10.1099/jgv.0.002293 Supplementary Material 1.

## Abbreviations

APN: aminopeptidase N
Co-IP: Co-immunoprecipitation
CoV: coronavirus
CPE: cytopathic effect
CSFV: classical swine fever virus
FMDV: foot-and-mouth disease virus
HEK293T: human embryonic kidney 293T
IFA: immunofluorescence assay
IFN-α: type I interferon alpha
IPEC: intestinal epithelial cell
ISG: interferon-stimulated gene
NC: nitrocellulose
Nsp12: nonstructural protein 12
PDCoV: porcine deltacoronavirus
PEDV: porcine epidemic diarrhoea virus
pMx1: porcine myxovirus resistance protein 1
PRRSV: porcine reproductive and respiratory syndrome virus
RdRp: RNA-dependent RNA polymerase
RTC: replication–transcription complex
RT-qPCR: real-time fluorescence quantitative PCR
siRNA: small interfering RNA
ST: swine testis
TGEV: transmissible gastroenteritis virus

## References

[1] Falchieri M, Coward VJ, Reid SM, Lewis T, Banyard AC (2024). Infectious bronchitis virus: an overview of the “chicken coronavirus”. J Med Microbiol.

[2] Cui J, Li F, Shi ZL (2019). Origin and evolution of pathogenic coronaviruses. Nat Rev Microbiol.

[3] He W-T, Ji X, He W, Dellicour S, Wang S (2020). Genomic epidemiology, evolution, and transmission dynamics of porcine deltacoronavirus. Mol Biol Evol.

[4] Hu H, Jung K, Vlasova AN, Chepngeno J, Lu Z (2015). Isolation and characterization of porcine deltacoronavirus from pigs with diarrhea in the United States. J Clin Microbiol.

[5] Ojkic D, Hazlett M, Fairles J, Marom A, Slavic D (2015). The first case of porcine epidemic diarrhea in Canada. Canadian Vet J.

[6] Saeng-Chuto K, Lorsirigool A, Temeeyasen G, Vui DT, Stott CJ (2017). Different lineage of porcine deltacoronavirus in Thailand, Vietnam and Lao PDR in 2015. Transbound Emerg Dis.

[7] Lee JH, Chung HC, Nguyen VG, Moon HJ, Kim HK (2016). Detection and phylogenetic analysis of porcine deltacoronavirus in Korean Swine Farms, 2015. Transbound Emerg Dis.

[8] Zhang J (2016). Porcine deltacoronavirus: overview of infection dynamics, diagnostic methods, prevalence and genetic evolution. Virus Res.

[9] Shan X, Li R, Ma X, Qiu G, Xiang Y (2024). Epidemiology, pathogenesis, immune evasion mechanism and vaccine development of porcine deltacoronavirus. Funct Integr Genomics.

[10] Niederwerder MC, Hesse RA (2018). Swine enteric coronavirus disease: A review of 4 years with porcine epidemic diarrhoea virus and porcine deltacoronavirus in the United States and Canada. Transbound Emerg Dis.

[11] Kong F, Wang Q, Kenney SP, Jung K, Vlasova AN (2022). Porcine deltacoronaviruses: origin, evolution, cross-species transmission and zoonotic potential. Pathogens.

[12] Turlewicz-Podbielska H, Pomorska-Mól M (2021). Porcine coronaviruses: overview of the state of the art. Virol Sin.

[13] Lee S, Lee C (2015). Functional characterization and proteomic analysis of the nucleocapsid protein of porcine deltacoronavirus. Virus Res.

[14] Ji W, Peng Q, Fang X, Li Z, Li Y (2022). Structures of a deltacoronavirus spike protein bound to porcine and human receptors. Nat Commun.

[15] Stanifer ML, Pervolaraki K, Boulant S (2019). Differential regulation of type I and type III interferon signaling. Int J Mol Sci.

[16] Yin L, Liu X, Hu D, Luo Y, Zhang G (2021). Swine enteric coronaviruses (PEDV, TGEV, and PDCoV) induce divergent interferon-stimulated gene responses and antigen presentation in porcine intestinal enteroids. Front Immunol.

[17] Huang C-H, Laurent-Rolle M, Grove TL, Hsu J-C (2025). Interferon-stimulated genes and immune metabolites as broad-spectrum biomarkers for viral infections. Viruses.

[18] Horisberger MA, Staeheli P, Haller O (1983). Interferon induces a unique protein in mouse cells bearing a gene for resistance to influenza virus. Proc Natl Acad Sci USA.

[19] Haller O, Staeheli P, Kochs G (2007). Interferon-induced Mx proteins in antiviral host defense. Biochimie.

[20] Verhelst J, Hulpiau P, Saelens X (2013). Mx proteins: antiviral gatekeepers that restrain the uninvited. Microbiol Mol Biol Rev.

[21] Haller O, Kochs G (2020). Mx genes: host determinants controlling influenza virus infection and trans-species transmission. Hum Genet.

[22] Bayrou C, Van Laere A-S, Dam Van P, Moula N, Garigliany M-M (2023). Anti-schmallenberg virus activities of type I/III interferons-induced mx1 gtpases from different mammalian species. Viruses.

[23] Dornfeld D, Petric PP, Hassan E, Zell R, Schwemmle M (2019). Eurasian avian-like swine influenza A viruses escape human MxA restriction through distinct mutations in their nucleoprotein. J Virol.

[24] Haller O, Staeheli P, Schwemmle M, Kochs G (2015). Mx GTPases: dynamin-like antiviral machines of innate immunity. Trends Microbiol.

[25] Zhou J, Chen J, Zhang X-M, Gao Z-C, Liu C-C (2018). Porcine Mx1 protein inhibits classical swine fever virus replication by targeting nonstructural protein NS5B. J Virol.

[26] Hu Y, Wu X, Tian Y, Jiang D, Ren J (2024). GTPase activity of porcine Mx1 plays a dominant role in inhibiting the N-Nsp9 interaction and thus inhibiting PRRSV replication. J Virol.

[27] Yuan B, Fang H, Shen C, Zheng C (2015). Expression of porcine Mx1 with FMDV IRES enhances the antiviral activity against foot-and-mouth disease virus in PK-15 cells. Arch Virol.

[28] He D, Zhang X, Liu K, Pang R, Zhao J (2014). *In vitro* inhibition of the replication of classical swine fever virus by porcine Mx1 protein. Antiviral Res.

[29] Wu J, Tang R, Zhang X, Gao M, Guo L (2024). IFITM3 restricts porcine deltacoronavirus infection by targeting its spike protein. Vet Microbiol.

[30] Jiang H, Jia M, Xiong J, Zhao C, Wang T (2024). The network interactions between the porcine deltacoronavirus nucleocapsid protein and host cellular proteins. Vet Microbiol.

[31] Zhang Q, Yoo D (2016). Immune evasion of porcine enteric coronaviruses and viral modulation of antiviral innate signaling. Virus Res.

[32] Huang H, Lei X, Zhao C, Qin Y, Li Y (2024). Porcine deltacoronavirus nsp5 antagonizes type I interferon signaling by cleaving IFIT3. J Virol.

[33] Liu S, Fang P, Ke W, Wang J, Wang X (2020). Porcine deltacoronavirus (PDCoV) infection antagonizes interferon-λ1 production. Vet Microbiol.

[34] Li Z, Tang W, Lai Y, Chen C, Fang P (2025). SIRT5-mediated desuccinylation of the porcine deltacoronavirus M protein drives pexophagy to enhance viral proliferation. PLOS Pathog.

[35] Zhang K, Lin S, Li J, Deng S, Zhang J (2022). Modulation of innate antiviral immune response by porcine enteric coronavirus. Front Microbiol.

[36] Sang Y, Rowland RRR, Hesse RA, Blecha F (2010). Differential expression and activity of the porcine type I interferon family. Physiol Genomics.

[37] Chang C, Sue S-C, Yu T, Hsieh C-M, Tsai C-K (2006). Modular organization of SARS coronavirus nucleocapsid protein. J Biomed Sci.

[38] Haller O, Stertz S, Kochs G (2007). The Mx GTPase family of interferon-induced antiviral proteins. Microbes Infect.

[39] Chen J, Hu J-H, Sun R-C, Li X-H, Zhou J (2023). Porcine Mx proteins inhibit pseudorabies virus replication through interfering with early gene synthesis. Vet Microbiol.

[40] Palm M, Garigliany MM, Cornet F, Desmecht D (2010). Interferon-induced Sus scrofa Mx1 blocks endocytic traffic of incoming influenza A virus particles. Vet Res.

[41] Zhang X, Jing J, Li W, Liu K, Shi B (2015). Porcine Mx1 fused to HIV Tat protein transduction domain (PTD) inhibits classical swine fever virus infection *in vitro* and *in vivo*. BMC Vet Res.

[42] Zhang X, He D-N, Zhou B, Pang R, Liu K (2013). *In vitro* inhibition of vesicular stomatitis virus replication by purified porcine Mx1 protein fused to HIV-1 Tat protein transduction domain (PTD). Antiviral Res.

[43] Ding Z, Luo S, Gong W, Wang L, Ding N (2020). Subcellular localization of the porcine deltacoronavirus nucleocapsid protein. Virus Genes.

[44] Lai MMC (1998). Cellular factors in the transcription and replication of viral RNA genomes: A parallel to DNA-dependent RNA transcription. Virology.

