# Supplementary material for: GTPase domain of porcine Mx1 protein interacts with the N protein of porcine deltacoronavirus to inhibit viral replication
Source: J Gen Virol. 2026 Aug 5;107(8):002293. doi: 10.1099/jgv.0.002293 (PMC13446539; doi:10.1099/jgv.0.002293)
Supplement: Supplementary file 1 [file jgv-107-02293-s001.pdf]

Supplementary Material

GTPase domain of porcine Mx1 protein interacts with the N protein of porcine deltacoronavirus to inhibit viral replication

Haikun Shangguan, Jianxiao Wu, Mingwei Li, Zhaoyang Ji, Yongrui Wang, Chunwei Zhang, Hexin Wang, Dailang Zhong, Zhihong Zhou, Xin Zhang, Da Shi, Hongyan Shi, Longjun Guo*, Li Feng*, Jianfei Chen*

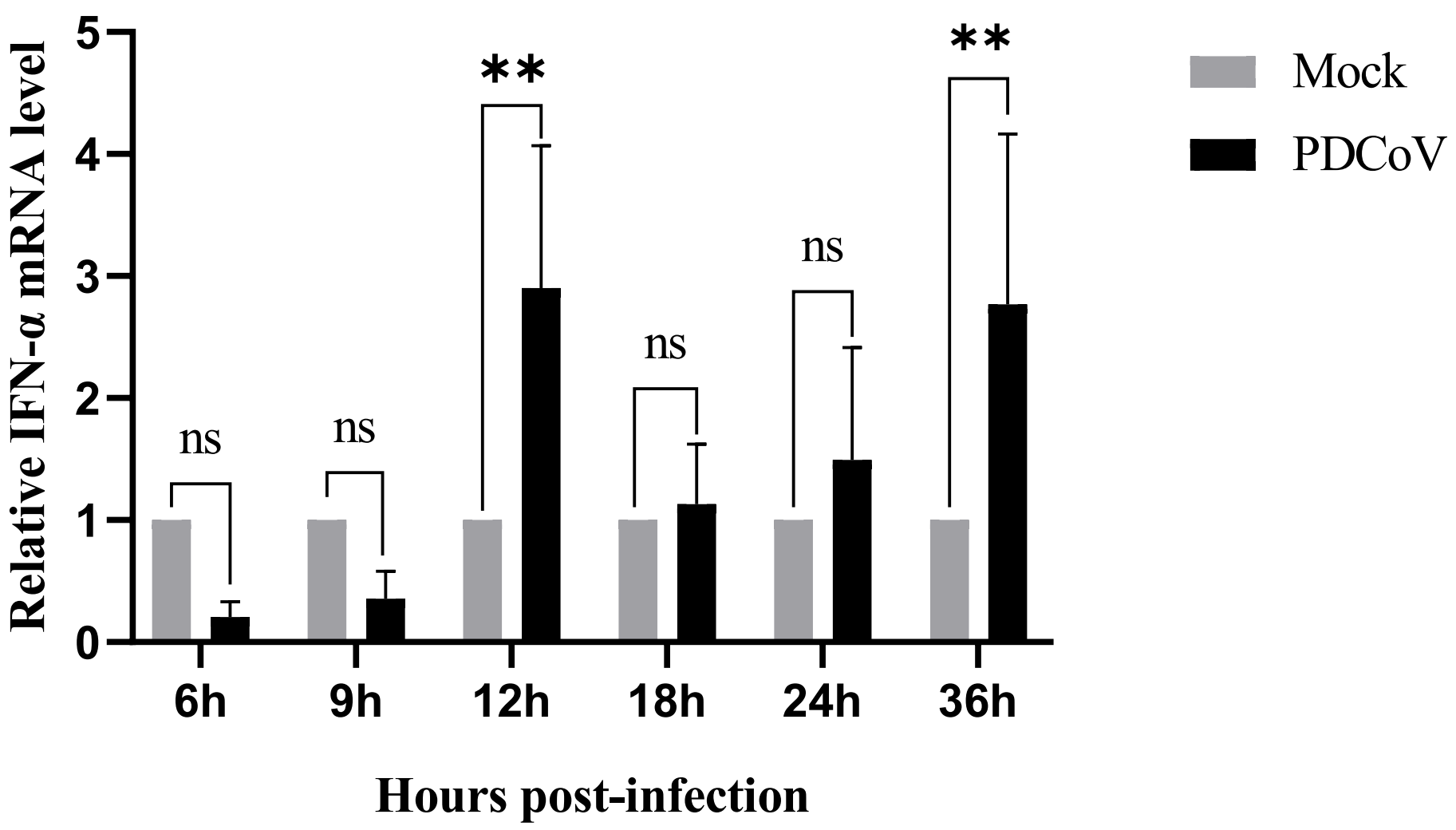

Figure S1. IPEC-J2 cells were infected with PDCoV at an MOI of 1.0, and cell lysates were collected at the indicated time points post-infection. Changes in IFN-α mRNA levels were determined by RT-qPCR. Data are presented as the mean ± SD of three independent experiments. Statistical significance was analyzed using two-way ANOVA followed by Bonferroni's post hoc test between PDCoV-infected groups and the mock controls. **, $P < 0.01$; ns, not significant.
